# Supplementary material for: Machine Learning Highlights Downtrending of COVID-19 Patients with a Distinct Laboratory Profile
Source: Health Data Sci. 2021 May 22;2021:7574903. doi: 10.34133/2021/7574903 (PMC9629663; doi:10.34133/2021/7574903)

**Supplemental Figure 1.** Distribution of representative laboratory tests of positive RT-PCR within the TPR, negative RT-PCR within the TPR, positive RT-PCR outside the TPR, and negative RT-PCR outside the TPR.


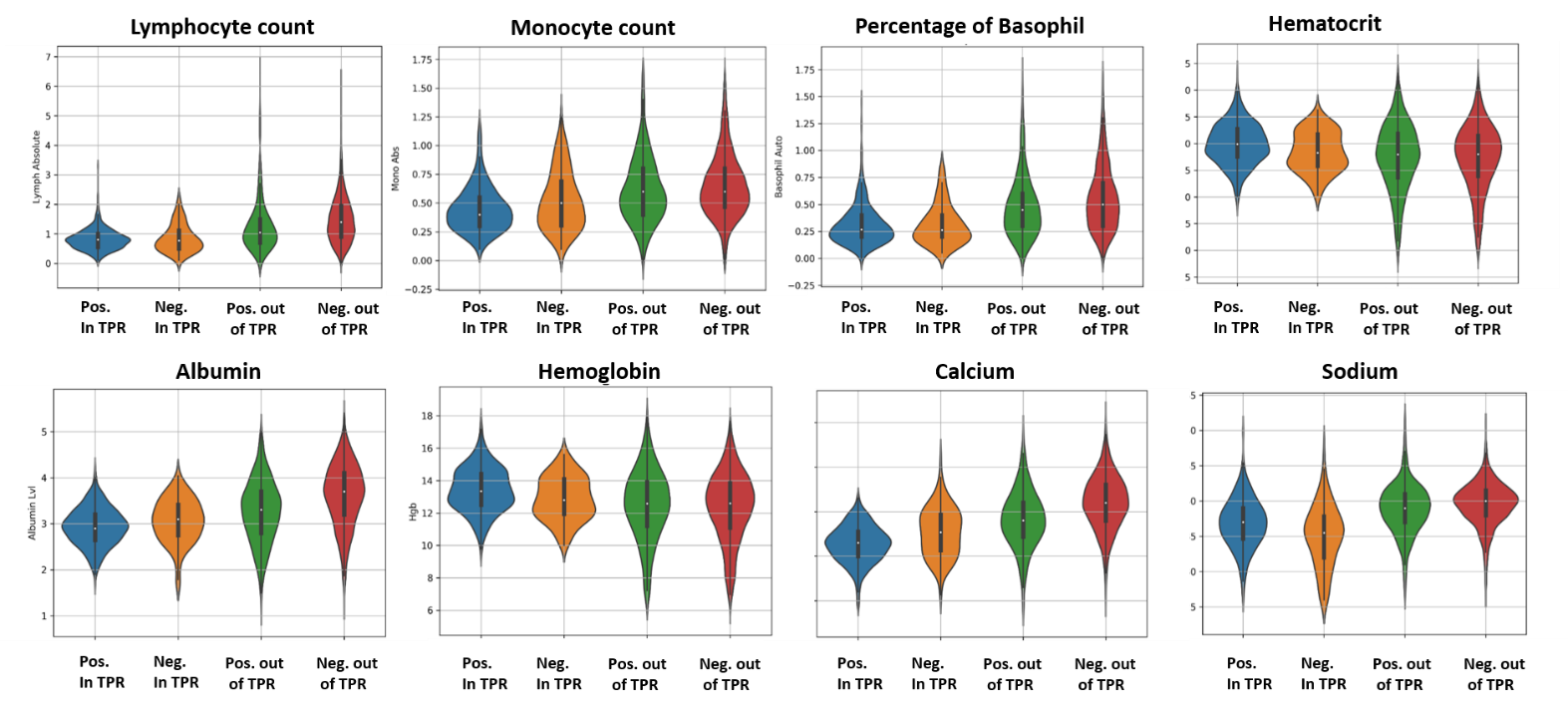

Supplement: Supplementary Materials — Supplemental Figure S1: distribution of representative laboratory tests of positive RT-PCR within the TPR, negative RT-PCR within the TPR, positive RT-PCR outside the TPR, and negative RT-PCR outside the TPR. [file 7574903.f1.docx]
